# Structural Characteristics of Mitochondrial Genomes of Eight Treehoppers (Hemiptera: Membracidae: Centrotinae) and Their Phylogenetic Implications

**DOI:** 10.3390/genes14071510

**Published:** 2023-07-24

**Authors:** Haijun Bai, Jinrui Zhang, Christopher H. Dietrich, Yiping Li, Xiangqun Yuan

**Affiliations:** 1 Key Laboratory of Plant Protection Resources and Pest Management, Ministry of Education, Entomological Museum, College of Plant Protection, Northwest A&F University, Yangling 712100, China; baihj@nwafu.edu.cn (H.B.); zhangjinrui@nwafu.edu.cn (J.Z.); 2 Illinois Natural History Survey, Prairie Research Institute, University of Illinois, Champaign, IL 61820, USA; chdietri@illinois.edu; 3 Key Laboratory of Integrated Pest Management on Crops in Northwestern Loess Plateau, Ministry of Agriculture, College of Plant Protection, Northwest A&F University, Yangling 712100, China

**Keywords:** mitogenome, phylogeny, phylogenetic analysis

## Abstract

Complete mitochondrial genomes were newly sequenced for eight species of the treehopper subfamily Centrotinae (Hemiptera: Membracidae), four of which represent genera for which mitogenomes were not previously available. The new mitogenomes are generally similar in overall structure, gene order, base composition, and nucleotide content to those of previously sequenced species of the subfamily. Phylogenetic analyses were conducted using both maximum likelihood and Bayesian inference methods based on three separate nucleotide sequence datasets in which RNA gene sequences and/or third codon positions were either included or excluded from the concatenated protein-coding gene alignments. The results are consistent with previous phylogenies based on morphology and partial nuclear genome data, except for the lack of support for the monophyly of Leptocentrini. These results show that mitogenome sequences are informative of both ancient and recent divergence patterns within Centrotinae.

## 1. Introduction

The insect mitogenome is a closed double-stranded biological macromolecule, typically with a length of 15–18 kb and containing 13 protein-coding genes (PCGs), 22 transfer RNAs (tRNAs), 2 ribosomal RNAs (rRNAs), and a non-coding region (NCR) [1,2]. Mitogenome sequence data have been widely used as molecular markers in the study of the taxonomy, systematics, and evolution of insects.

Treehoppers (Membracidae) are one of the most speciose families of the order Hemiptera and well known for their morphological novelties, particularly an enlarged and often highly ornamented pronotum [3,4,5,6,7,8]. Centrotinae is the largest subfamily of Membracidae and the only one that is globally distributed, comprising over 1300 species from 216 recorded genera [8]. Despite its richness, the phylogeny of Centrotinae remains minimally explored. Previous research mainly focused on descriptions of new species and morphology-based taxonomy [5,6,9,10,11,12,13]. Only a few phylogenetic studies have been conducted, most notably the comprehensive morphology-based study of Wallace and Deitz (2004) [14]. More recent phylogenomic analyses of Membracidae [15,16] strongly supported the monophyly of Centrotinae and resolved relationships among some major lineages but included only 14 and 10 representatives of the subfamily, respectively. Previous studies by Hu et al. (2019) [17] and Yu et al. (2022) [18] yielded completed mitogenome sequences for eight species of this subfamily, and a recent phylotranscriptomic study provided potential data for ten species [16]. Until now, fewer than 20 mitogenomes of Centrotinae have been available in the NCBI. The systematics of Centrotinae are of great significance to the control of economic plant pests and the protection and utilization of biological resources for four main reasons: (1) some species can causes apple and other fruit trees to wilt by laying eggs in the twigs, some may infest soybeans with such large populations that ovipositional scars can impact yields, and some may cause similar damage in avocados [19,20]; (2) Centrotinae is an important pest, as it sucks plant sap and is an important carrier of plant pathogens; (3) it is an ideal material to study the social development of insects with profemale egg protection behavior and presocial social behavior of nymphs; and (4) it has a global distribution and is of great significance for the study of biogeography [6,8,14].

In this contribution, we provide a more comprehensive molecular phylogenetic analysis of Centrotinae, combining eight newly determined mitogenomes (including four previously unrepresented genera) and previously available public data to infer a robust evolutionary framework. Our results provide new insights into the phylogeny (especially at the tribe and genus levels) of this diverse group.

## 2. Materials and Methods

### 2.1. Sample Collection and DNA Extraction

The collection locations of adult specimens of the eight species, namely *Antialcidas trifoliaceus* (Walker, 1858), *Nondenticentrus paramelanicus* (Zhang et Yuan, 1998), *Pantaleon erectonodatus* (Chou et Yuan, 1983), *Tribulocentrus zhenbaensis* (Chou et Yuan, 1982), *Leptobelus boreosinensis* (Yuan et Chou, 1988), *Hemicentrus obliquus* (Yuan et Tian, 1994), *Leptocentrus formosanus* (Matsumura, 1912), and *Leptocentrus longispinus* (Distant, 1907) are provided in Table S1. All materials were preserved in 100% ethanol immediately after collection and kept at −20 °C in the Entomological Museum of Northwest A&F University, Yangling, Shaanxi Province, China. Specimen identification was based on morphological characteristics. Genomic DNA was isolated from the thoracic tissue using an EasyPure^®^ Genomic DNA Kit (TransGen Biotech, Beijing, China).

### 2.2. Sequencing, Assembly, Annotation, and Bioinformatic Analyses

The complete mitogenomes of eight species were sequenced using next-generation sequencing on the Illumina HiSeq 2000 platform (Novogene, Beijing, China). The circular mitogenomes were assembled using GetOrganelle [21] from paired-end raw data. The orientation, size, and position of each gene were predicted on the MITOS Web Server [22], and the open reading frames (ORFs) of PCGs were corrected manually using Geneious based on the invertebrate codon table (the 5th codon table) (Biomatters, Auckland, New Zealand). The secondary structures of tRNAs were manually plotted using Adobe Illustrator CS5. The nucleotide composition and codon usage of the eight mitogenomes were calculated and analyzed using PhyloSuite [23], and the data obtained from this analysis were utilized to generate RSCU (relative synonymous codon usage) figures. AT-skew and GC-skew were computed according to the following formulas: AT-skew = [A − T]/[A + T] and GC-skew = [G − C]/[G + C]. The complete sequences of all 8 species were uploaded to GenBank with accession numbers OQ984256–OQ984263.

### 2.3. Analysis of Codon Usage Bias

The *Nĉ* values, indicating the “effective number of codons used in a gene”, were calculated for 13 PCGs using CodonW software (version 1.1.4, created by John Peden, Nottingham, England). The value of *Nĉ* can range from 20 in the case of a strong bias where one codon is exclusively used for each amino acid to 61 when the usage of alternative synonymous codons is equally probable [24], and Genes with *Nĉ* values below 35 are considered to exhibit significant codon bias [25].

The GC-bias and AT-bias values are measured in [G3/(G3 + C3)] and [A3/(A3 + T3)], respectively [26]. In a Parity rule 2 (PR2) plot, the AT-bias value at the third codon position of four-codon amino acids is represented on the *y*-axis, while the GC-bias value is represented on the *x*-axis. The center of the plot, where both coordinates are 0.5, corresponds to A = U and G = C (PR2), indicating no bias resulting from the influence of the mutation and selection rates. The PR2 plot was drawn using GraphPad Prism.

### 2.4. Phylogenetic Analysis

The taxon sampled 24 species of the subfamily Centrotinae (8 newly sequenced in this study, 16 available from GenBank), representing 5 tribes and 15 genera. Two species of the subfamily Smiliinae, *Entylia carinata* (NCBI accession number: NC_033539) and *Stictocephala bisonia* (NC_057522), were selected as outgroups. Data for included samples are provided in Table S1.

The sequences were processed as follows. All individual genes were aligned using MAFFT with the L-INS-i strategy [27,28]. Alignment trimming was performed using trimAl [29]. Then, all individual genes were concatenated into 3 sub-datasets for phylogenetic analysis: (1) PCG matrix, containing all codon positions of all 13 protein-coding genes; (2) P123RT matrix, concatenating the PCG matrix and 24 RNA genes (including 22 tRNAs and 2 rRNAs); and (3) P12RT matrix, removing the third codon positions of the 13 PCGs of the P123RT matrix. Based on these 3 datasets, phylogenetic trees were reconstructed using both maximum likelihood (ML) and Bayesian inference (BI) methods. The optimal partition schemes and nucleotide substitution models were analyzed using PartitionFinder2 [30] (Table S1). ML analyses were conducted using IQ-TREE [31] with the ultrafast bootstrap (UFB) approximation approach [32]. The support value of each node is based on 10,000 bootstrap replicates. BI analyses were inferred from MrBayes [33]. Two independent runs of 2 × 10^7^ generations were conducted with four independent Markov Chain Monte Carlo (MCMC) chains, sampling every 1000 generations. When the average standard deviation of split frequencies fell below 0.01, the run was assumed to be convergent. A consensus tree was generated from all the trees after discarding the first 25% of trees from each MCMC run as burn-in. The support value of each node is represented as Bayesian posterior probability (BPP).

## 3. Results and Discussion

### 3.1. Genome Structure and Organization

All eight genomes of the eight studied species consisted of a typical structure with 13 PCGs, 22 tRNA genes, 2 rRNA genes, and a major non-coding A + T-rich region, which is considered to be the replication initiation site. The lengths of the mitogenomes varied among the eight horned species. The full lengths of *A. trifoliaceus*, *H. obliquus*, *L. boreosinensis*, *L. formosanus*, *L. longispinus*, *N. paramelanicus*, *P. erectonodatus*, and *T. zhenbaensis* are 15,249 bp, 15,570 bp, 15,045 bp, 15,399 bp, 15,323 bp, 15,804 bp, 15,747 bp, and 16,598 bp, respectively. *T. zhenbaensis* has the longest genome, while *L. boreosinensis* is the shortest (Table S3).

Among the eight species of Centrotinae, the base A accounted for 43.5%~44.8% (*A. trifoliaceus* and *T. zhenbaensis* were both 43.5%), C accounted for 12.3%~14.2%, T accounted for 32.5%~34.4% (*H. obliquus* and *L. boreosinensis* were both 32.5%), the proportion of G was 8.6%~9.9% (*H. obliquus* and *P. erectonodatus* were both 9.6%), and the content of A + T was 76.7%~78.3% (Table S3).

### 3.2. Protein-Coding Genes (PCGs) and Codon Usage

**Figure 1:**
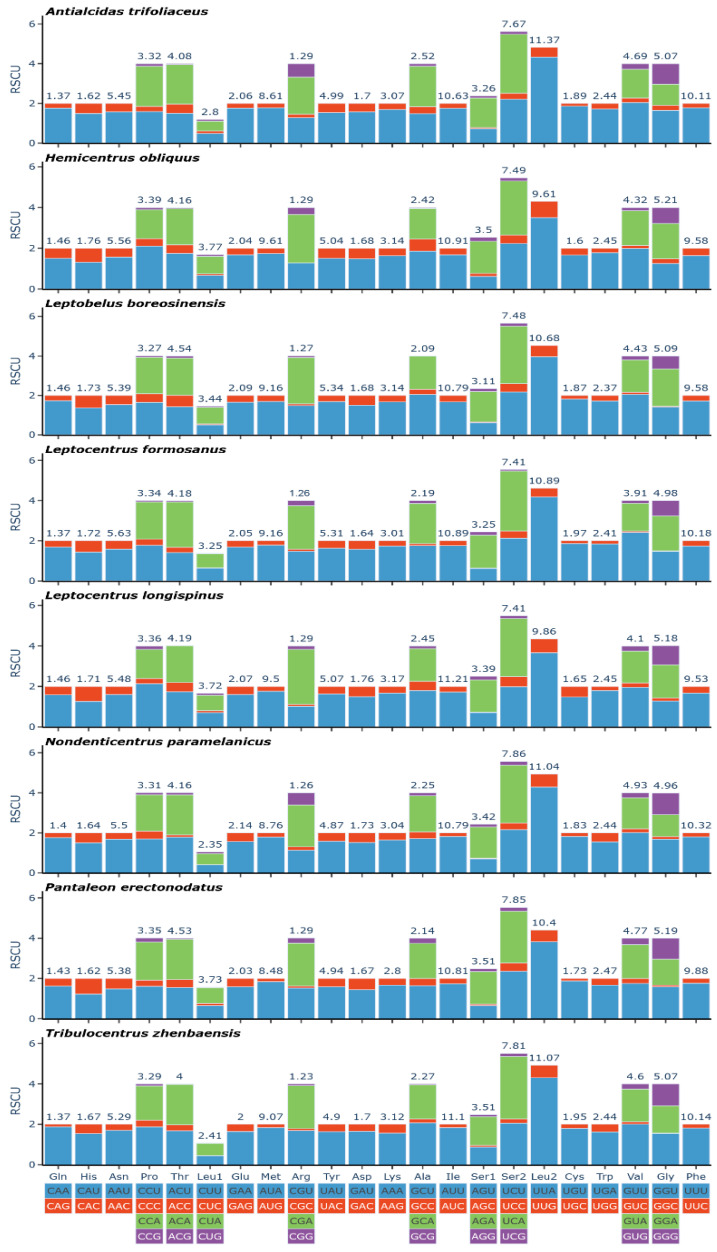
Relative synonymous codon usage (RSCU) in the mitogenomes of eight treehoppers.

The lengths of 13 PCGs of 8 species of Centrotinae were 10,926 bp~11,004 bp, with *H. obliquus* and *L. longispinus* representing the shortest PCGs and *L. formosanus* representing the longest PCG. Among the eight sequenced species, 9 of the 13 PCGs (*nad2*, *cox1*, *cox2*, *atp8*, *atp6*, *cox3*, *nad3*, *nad6*, and *Cytb*) were encoded on the J strand, and the other 4 (*nad5*, *nad4*, *nad4L*, and *nad1*) were encoded on the N strand. Among the 13 PCGs, the smallest gene was *atp8*, and the largest gene was *nad5*, ranging from 153 to 1692 bp. In addition, T content in PCGs of the eight sequenced species was the highest, and A + T content was significantly higher than that of C + G (Table S3). The AT content of the third codon (85.5%~88.2%) was significantly higher than that of the first codon positions (72.8%~74.5%) and the second positions (68%~69.1%) (Table S3). All PCGs of the eight species were terminated with TTA, TAG, or single T, and *cox2* and *nad5* ended with single T most frequently, while *Cytb*, *nad4*, *nad4L*, and *nad5* occasionally ended with TAG (Table S4). Statistics on the RSCU show that UUA (Leu2) and UCA (Ser2) are the two most frequently used codons (Figure 1).

### 3.3. Nucleotide Diversity in PCGs of Mitogenomes

**Figure 2:**
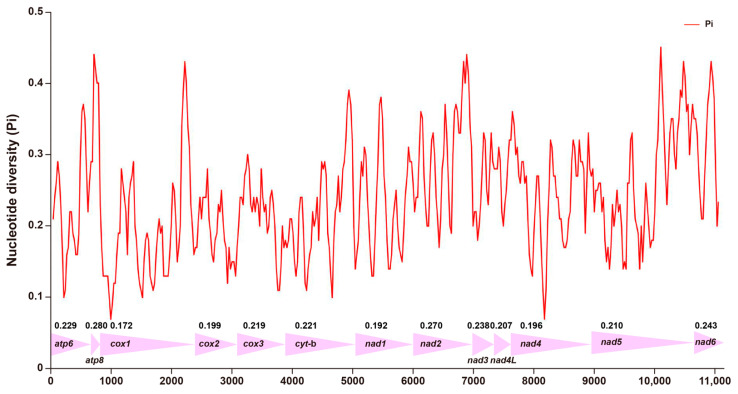
Sliding window analysis of 13 PCGs based on 8 species. The red line shows the value of nucleotide diversity (Pi). The Pi value of each gene is shown in the graph.

The nucleotide diversity (Pi values) of 13 PCGs in the mitogenomes was analyzed using DnaSP software [34]. The nucleotides varied greatly among different genes (Figure 2). The nucleotide diversity values ranged from 0.172 (*cox1*) to 0.280 (*atp8*). The *atp6*, *atp8*, *cox3*, *cytb*, *nad2*, *nad3*, *nad4L*, *nad5 and nad6* Pi values above 0.2 are 0.229, 0.280, 0.219, 0.221, 0.270, 0.238, 0.207, 0.210, and 0.243, respectively. The nucleotide diversity values of *cox1*, *cox2*, *nad1*, and *nad4* were relatively low (Pi = 0.172, 0.199, 0.192, and 0.196, respectively), corresponding to relatively conserved genes in the 13 PCGs (Figure 2).

### 3.4. Analysis of Codon Usage Bias

The *Nĉ* values of 24 Centrotinae species ranged from 35.420 (*Machaerotypus stigmosus*, Gargarini) to 41.840 (*Tricentrus gammamaculatus*, Gargarini) (all > 35) (Table S1), indicating that the concatenated 13 PCGs exhibit no significant codon bias. And also found no obvious regularity in the *Nĉ* values of species within different tribes. In addition, two species, *Hypsauchenia hardwickii* (Hypsauchenini) and *Antialcidas floripennae* (Gargarini), had minimum and maximum GC and GC12 values, respectively (Table S2). GC3 values ranged from 0.116 (*Gargara genistae*, Gargarini) to 0.188 (*A. floripennae*, Gargarini). The codon features of 13 individual PCGs are shown in Table S5.

**Figure 3:**
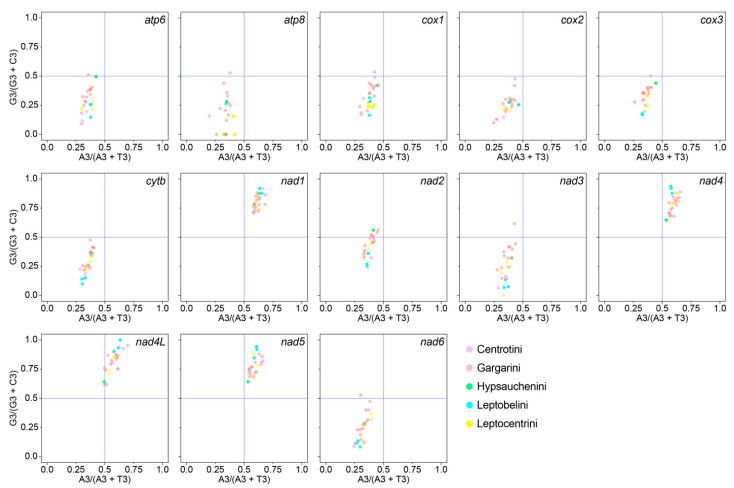
PR2 plot of 13 PCGs of 24 Centrotinae species. The AT-bias value at the third codon position of four-codon amino acids is represented on the *y*-axis, while the GC-bias value is represented on the *x*-axis. Five tribes, Centrotini (2 spp.), Gargarini (14 spp.), Hypsauchenini (1 sp.), Leptobelini (3 spp.), and Leptocentrini (4 spp.), are marked in different colors.

To examine whether codon bias resulted from mutation pressure or natural selection, a PR2 plot was utilized to analyze the relationship between the G and C contents, as well as the relationship between the A and T contents, in the 13 PCGs (Figure 3). We reported that the codon usage bias of all PCG codons was influenced by both natural selection and mutation pressure. In addition, the distribution of distinct tribes of each gene was irregular (Figure 3), which may indicate a lack of regularity in mutation and selection pressure across taxa.

### 3.5. Ribosomal and Transfer RNA Genes

Among the 22 tRNA genes, 14 were encoded on the J strand, and 8 were encoded on the N strand, with lengths of 60~70 bp and a total length of 1402 bp~1440 bp. The contents of A (39.5%~41.1%) and T (37.8%~39.6%) were similar, and the content of G (39.5%~40.9%) was significantly higher than that of C (8.1%~9.1%). The content of A + T in tRNA was 78.7%~79.6%, which is slightly higher than that in PCGs. The AT skew value ranged between −0.001 and 0.039, and GC-skew range between 0.144 and 0.21 (Table S3). All tRNA genes can be folded into typical cloverleaf secondary structures, except for *trnS* (AGN), which lacks a DHU arm (Figures S1–S8). The total lengths of the two rRNA genes (*rrnL* and *rrnS*) encoded by the N strand is 1914 bp~1926 bp (Table S4). The content of A is 31.1%~33.5%, the content of T is 46.6%~50.4%, the content of C is 6.5%~7.5%, and the content of G is 11.6%~13.3%. AT skew ranges between −0.238 and −0.163, and GC-skew ranges between 0.216 and 0.274 (Table S3).

### 3.6. Overlapping Sequences and Intergenic Spacers

An overlapping gene (or OLG) is a gene whose expressible nucleotide sequence partially overlaps with the expressible nucleotide sequence of another gene [35]. Spacer DNA is the non-coding DNA region between genes [36,37]. *A. trifoliaceus*, *H. obliquus*, *L. boreosinensis*, *L. formosanus*, *L. longispinus*, *N. paramelanicus*, *P. erectonodatus*, and *T. zhenbaensis* contained 17, 17, 18, 19, 18, 19, 19, and 19 overlapping genes, respectively. The longest overlap, 39 bp (*nad6-Cytb*), occurs in the mitogenome of *A. trifoliaceus*; *L. boreosinensis* is 15 bp (*trnM-nad2*), *L. formosanus* is 20 bp *(trnF-nad5* and *nad6-Cytb*), *L. longispinus is* 8 bp (*nad6-Cytb*), *N. paramelanicus* is 29 bp (*nad6-Cytb*), *P. erectonodatus* is 26 bp (*trnF-nad5*), and *T. zhenbaensis* is also 26 bp (*trnF-nad5*), containing 7, 8, 10, 5, 6, 8, 8, and 8 intergenic spacers, respectively. The size of a spacer is generally 1~7 bp, and only one very long intergenic spacer of 130 bp existed only in *P. erectonodatus* between *nad4L* and *trnT* (Table S4).

### 3.7. A + T-Rich Region

The A + T-rich region is the site where a replication complex is formed and where DNA synthesis is initiated [36]. Of the eight species investigated in this study, the length of the A + T-rich region ranged from 783 bp to 2384 bp, with *L. boreosinensis* being the shortest and *T. zhenbaensis* being the longest. In the A + T-rich region, the A content ranged from 44% to 46.4%. *A. trifoliaceus* contained the lowest amount of A, and *L. longispinus* contained the highest amount of A. The T content ranged from 36.4% to 40.3%. The lowest amount was found in *T. zhenbaensis*, and the highest content was found in *A. trifoliaceus.* C content ranged from 5.6% to 10.8%, and *H. obliquus* and *T. zhenbaensis* had the lowest amount and the highest amount, respectively. The G content ranged from 7.5% to 11.5%, and the contents of *L. formosanus* and *T. zhenbaensis* were the lowest and the highest, respectively (Table S3).

### 3.8. Phylogenetic Relationships

**Figure 4:**
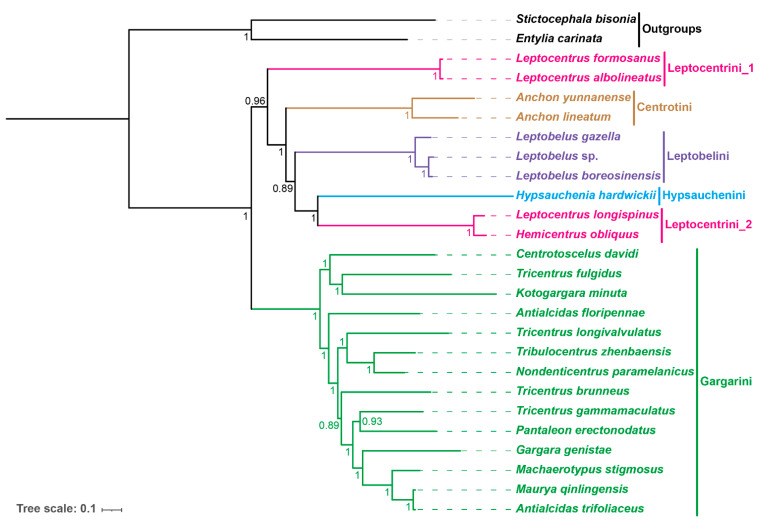
Circular map of the mitochondrial genome of eight species and phylogenetic relationships of the subfamily Centrotinae inferred by a BI method based on the P123RT dataset. Numbers on nodes are the posterior probabilities (BPP).

Phylogenetic analyses performed using ML and BI methods yielded similar topologies for the same sub-datasets (PCG matrix, P123RT matrix, and P12RT matrix), but topologies based on different datasets differed (see Figures S9–S13). Specifically, the intertribe relationships of the PCG and P123RT datasets are consistent with the topology ((Leptocentrini_1 + (Centrotini + (Leptobelini + (Leptocentrini_2 + Hypsauchenini)))) + Gargarini) (Figure 4, Figures S9, S10 and S13), while distinct from that based on P12RT datasets ((Leptocentrini_1 + ((Leptocentrini_2 + Hypsauchenini) + (Centrotini + Leptobelini))) + Gargarini) (Figures S11 and S12). Our results show that differences among datasets significantly contribute to phylogenetic inconsistencies. Despite this, the P123RT dataset appears to be more highly supported based on the stronger overall branch support. For simplicity and brevity, only the BI result for this dataset is presented here (Figure 4).

The phylogenetic tree consists of six major clades corresponding to five tribes (Figure 4). The phylogeny is consistent with the morphology-based tribal classification of Wallace and Deitz (2004) [14], except the four included representatives of Leptocentrini are divided among two independent clades. Considering our molecular evidence suggesting that this tribe may be a polyphyletic group, some morphological characteristics are provided. The humeral angle of the Leptocentrini species is developed, and two species of Leptocentrin_1 are triangular. In addition, the prothorax bevel of the two species of Leptocentrin_1 are slightly inclined, but that of the two species of Leptocentrin_2 is completely vertical. In addition, the frontoclypeu lateral flap of both species of Leptocentrin_2 is obvious, whereas that of *L. albolineatus* is not.

The genera *Antialcidas*, *Leptocentrus*, and *Tricentrus* are polyphyletic. This is not surprising because the latter two are very large and poorly defined morphologically. This result also agrees with the phylogeny of Wallace and Deitz (2004) [14] in showing that *Hemicentrus*, which lacks a posterior pronotal process, evolved from within a lineage that has the posterior pronotal process normally developed and extended over the scutellum.

Our mitogenome-based phylogeny is also consistent with the morphology-based phylogeny of Wallace and Deitz (2004) [14] and the phylogenomic analysis of Dietrich et al. (2017) [15] in placing Gargarini in a clade sister to the clade comprising Leptocentrini, Centrotini, Leptobelini, and Hypsaucheniini. Within the latter, relationships among tribes are generally consistent with those reported previous studies, with the exception of the polyphyly of Leptocentrini inferred in our study. No previous molecular phylogenetic study has included more than one representative of this tribe, so its monophyly has not been tested previously using molecular data. Nevertheless, the intertribe relationships reported in this and previous mitogenome-based studies remain inconsistent, which may be the result of sampling bias or insufficient sampling [38,39]. Studies incorporating representatives of additional genera and tribes are needed.

Collectively, this study yielded a robust phylogeny with high support of all internal nodes (BPP = 0.89~1) and generally congruent with prior analyses based on morphology and other genomic data. According to a previous molecular time tree, the diversification of tribes within this group began at least 47 million years ago [15]. This indicates that mitogenome sequences are informative of both ancient and recent phylogenetic splits within Centrotinae.

## Data Availability

The complete mitochondrial genomes of *Antialcidas trifoliaceus*, *Hemicentrus obliquus*, *Leptobelus boreosinensis*, *Leptocentrus formosanus*, *Leptocentrus longispinus*, *Nondenticentrus paramelanicus*, *Pantaleon erectonodatus*, and *Tribulocentrus zhenbaensis* are deposited in the GenBank of the NCBI under accession numbers OQ984256–OQ984263, respectively.

## Supplementary Materials

The following supporting information can be downloaded at: https://www.mdpi.com/article/10.3390/genes14071510/s1, Figure S1: Predicted secondary cloverleaf structure for the tRNA genes of *Antialcidas trifoliaceus*; Figure S2: Predicted secondary cloverleaf structure for the tRNA genes of *Hemicentrus obliquus*; Figure S3: Predicted secondary cloverleaf structure for the tRNA genes of *Leptobelus boreosinensis*; Figure S4: Predicted secondary cloverleaf structure for the tRNA genes of *Leptocentrus formosanus*; Figure S5: Predicted secondary cloverleaf structure for the tRNA genes of *Leptocentrus longispinus*; Figure S6: Predicted secondary cloverleaf structure for the tRNA genes of *Nondenticentrus paramelanicus*; Figure S7: Predicted secondary cloverleaf structure for the tRNA genes of *Pantaleon erectonodatus*; Figure S8: Predicted secondary cloverleaf structure for the tRNA genes of *Tribulocentrus zhenbaensis*; Figure S9: Phylogenetic tree inferred by ML method based on PCG dataset. Numbers on nodes are the bootstrap support values (BS); Figure S10: Phylogenetic tree inferred by BI method based on PCG dataset. Numbers on nodes are the posterior probabilities (BPP); Figure S11: Phylogenetic tree inferred by ML method based on P12RT dataset. Numbers on nodes are the bootstrap support values (BS); Figure S12: Phylogenetic tree inferred by BI method based on P12RT dataset. Numbers on nodes are the posterior probabilities (BPP); Figure S13: Phylogenetic tree inferred by ML method based on P123RT dataset. Numbers on nodes are the bootstrap support values (BS); Table S1: The species information (References [17,18,40,41,42,43,44] are cited in the supplementary materials) and codon features of PCGs of 24 Centrotinae species and 2 outgroups used in phylogenetic analyses. Notably, GC indicates the GC average content of three codon positions; GC12 indicates the GC content of the first and second positions of all codons in the gene, and GC3, A3, T3, G3, and G3 are the same under analogy; Table S2: Best partitioning schemes and models based on different datasets; Table S3: Nucleotide composition and skewness of different elements of mitogenomes *Antialcidas trifoliaceus*/*Hemicentrus obliquus*/*Leptobelus boreosinensis*/*Leptocentrus formosanus*/*Leptocentrus longispinus*/*Nondenticentrus paramelanicus*/*Pantaleon erectonodatus*/*Tribulocentrus zhenbaensis*; Table S4: Mitogenomic organization of *Antialcidas trifoliaceus*/*Hemicentrus obliquus*/*Leptobelus boreosinensis*/*Leptocentrus formosanus*/*Leptocentrus longispinus*/*Nondenticentrus paramelanicus*/*Pantaleon erectonodatus*/*Tribulocentrus zhenbaensis*; Table S5: Codon features of 13 PCGs of 24 Centrotinae species. Notably, the *Nĉ* values of the *atp8* gene in some species were missing because the sequence length was too short to contain amino acids with four synonymous codons.
