# Supplementary material for: Structural Characteristics of Mitochondrial Genomes of Eight Treehoppers (Hemiptera: Membracidae: Centrotinae) and Their Phylogenetic Implications
Source: Genes (Basel). 2023 Jul 24;14(7):1510. doi: 10.3390/genes14071510 (PMC10379000; doi:10.3390/genes14071510)
Supplement: Supplementary file 1 [file genes-14-01510-s001.zip › Supplementary_files/Supplementary_figures.pdf]

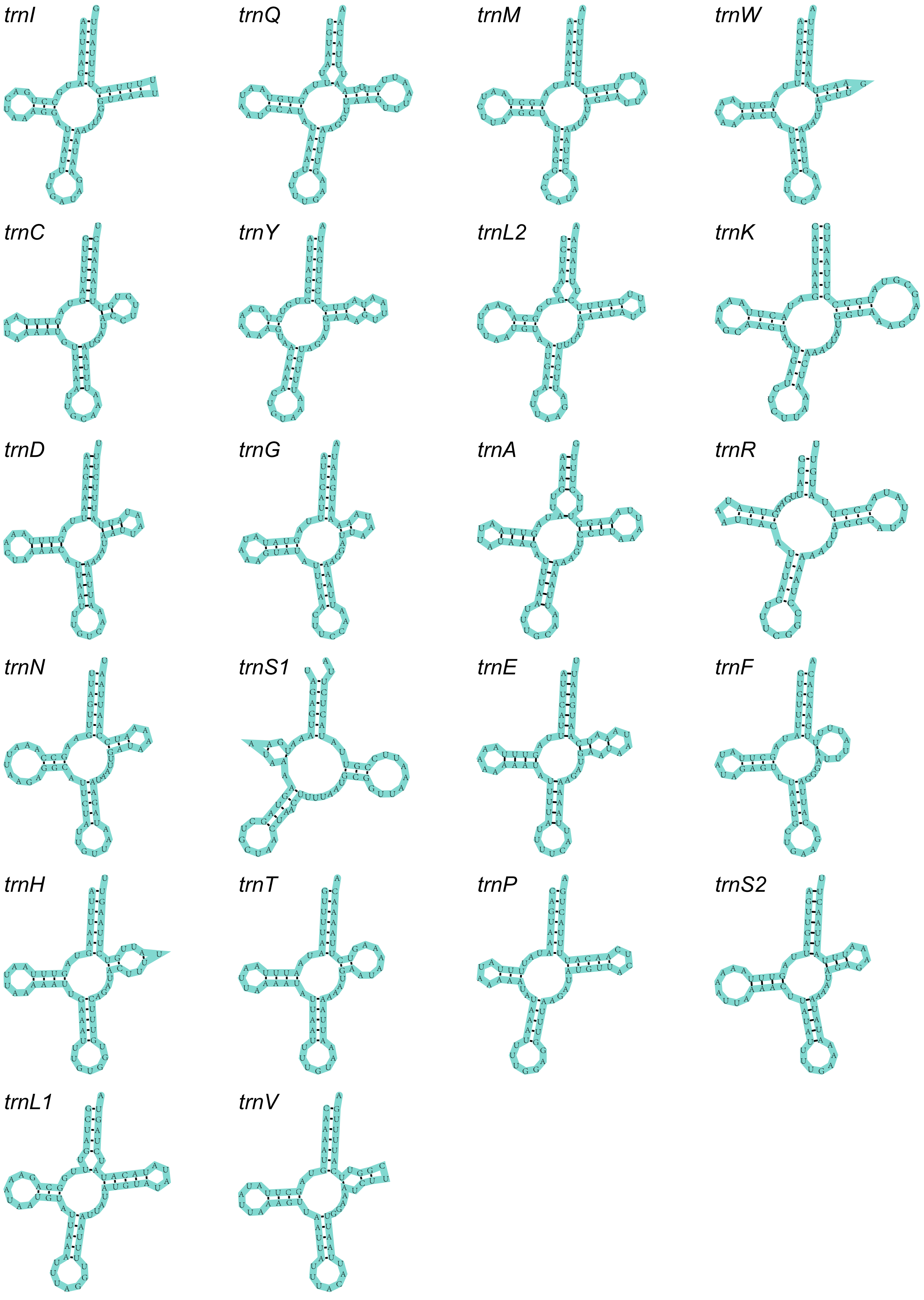

Figure S1. Predicted secondary clover-leaf structure for the tRNA genes of *Antialcidas trifoliaceus*.

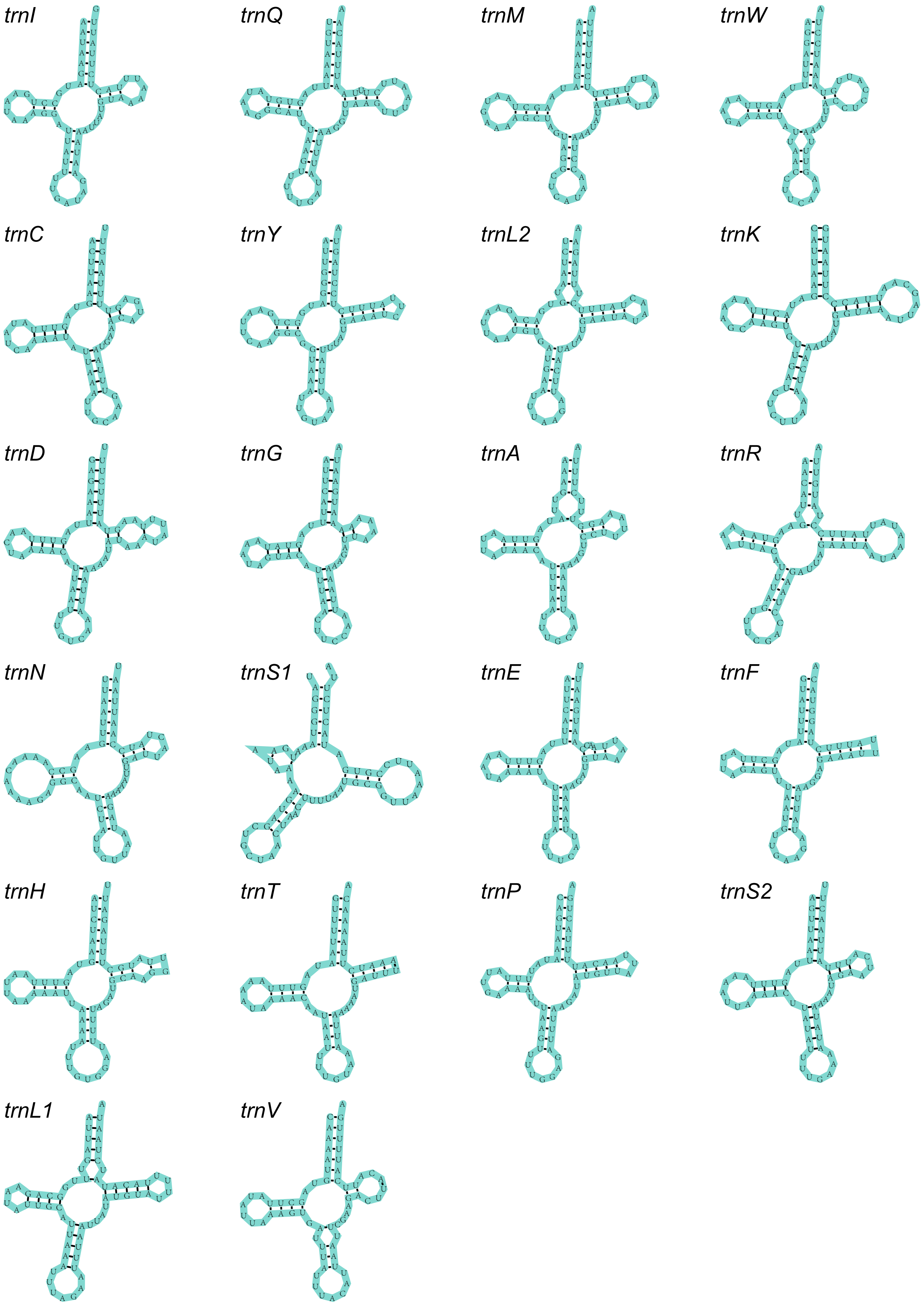

Figure S2. Predicted secondary clover-leaf structure for the tRNA genes of *Hemicentrus obliquus*.

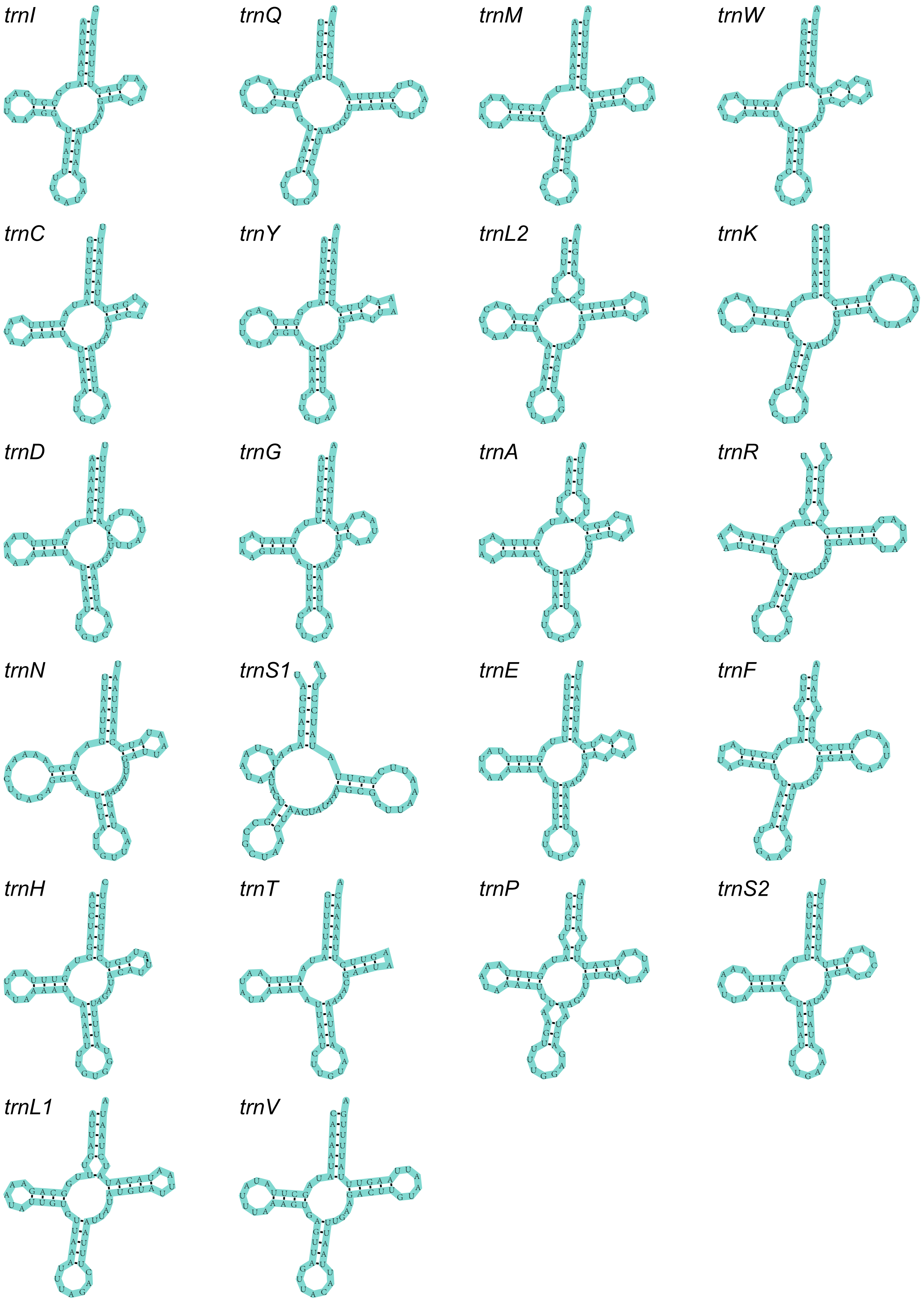

Figure S3. Predicted secondary clover-leaf structure for the tRNA genes of *Leptobelus boreosinensis*.

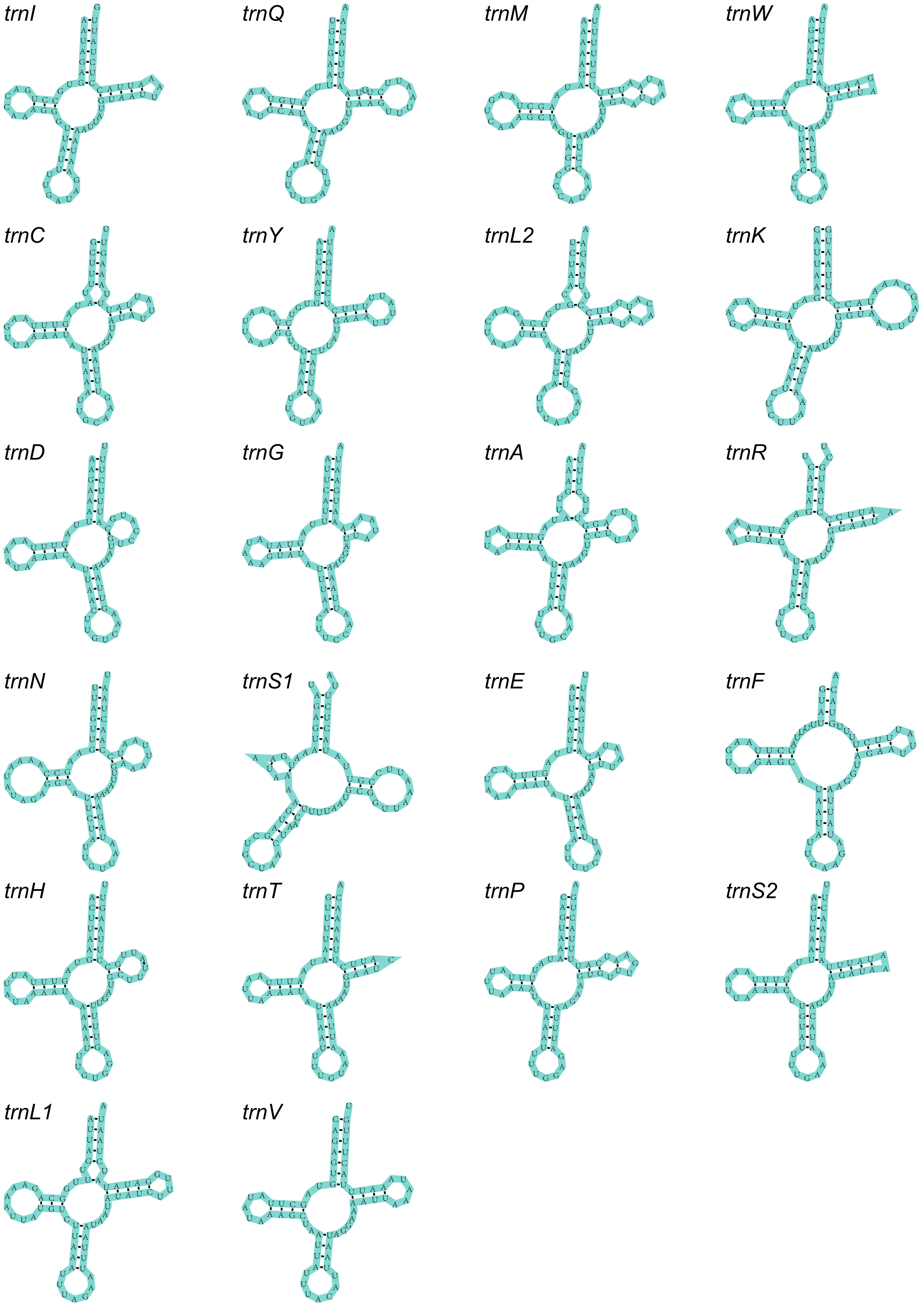

Figure S4. Predicted secondary clover-leaf structure for the tRNA genes of *Leptocentrus formosanus*.

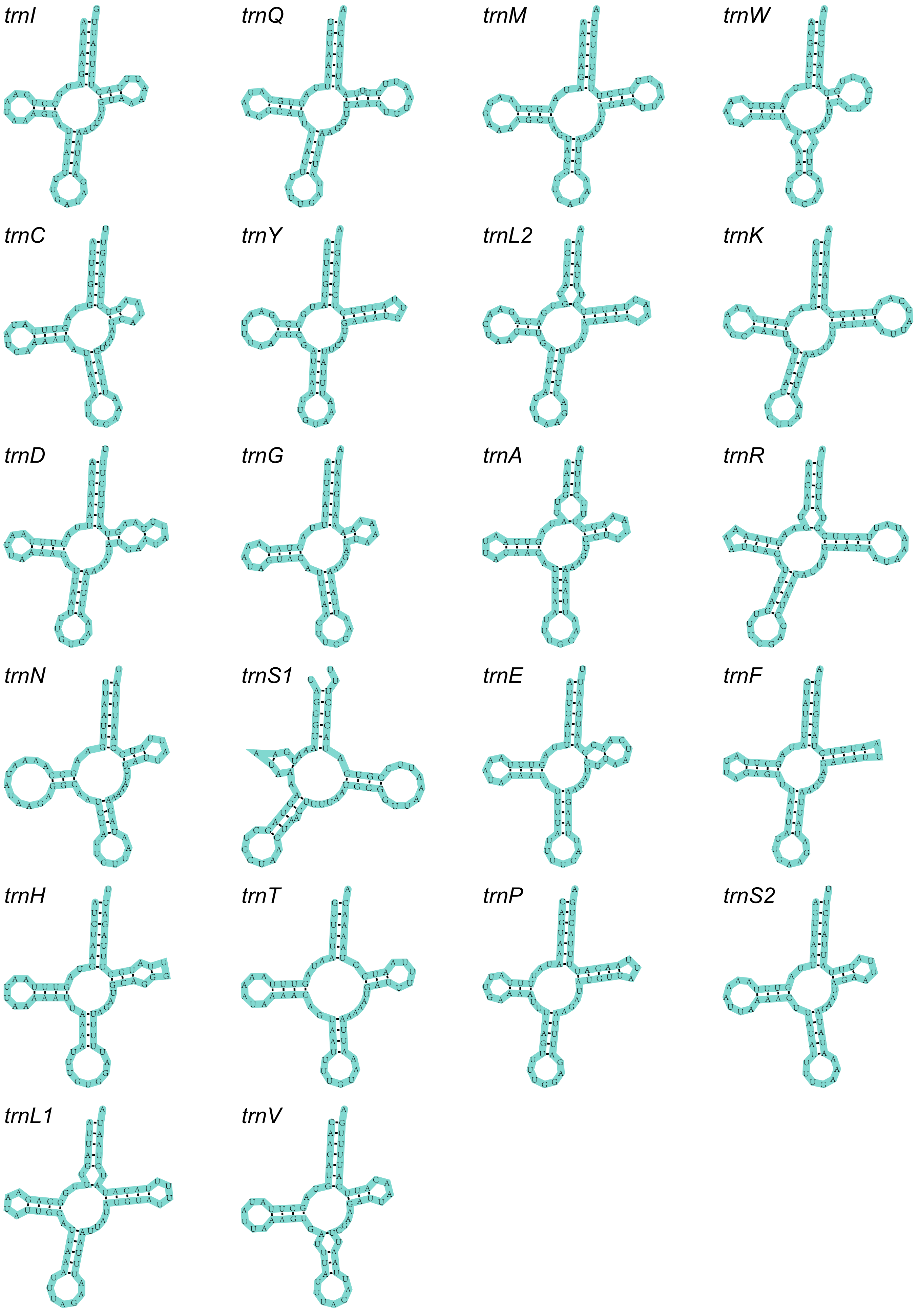

Figure S5. Predicted secondary clover-leaf structure for the tRNA genes of *Leptocentrus longispinus*.

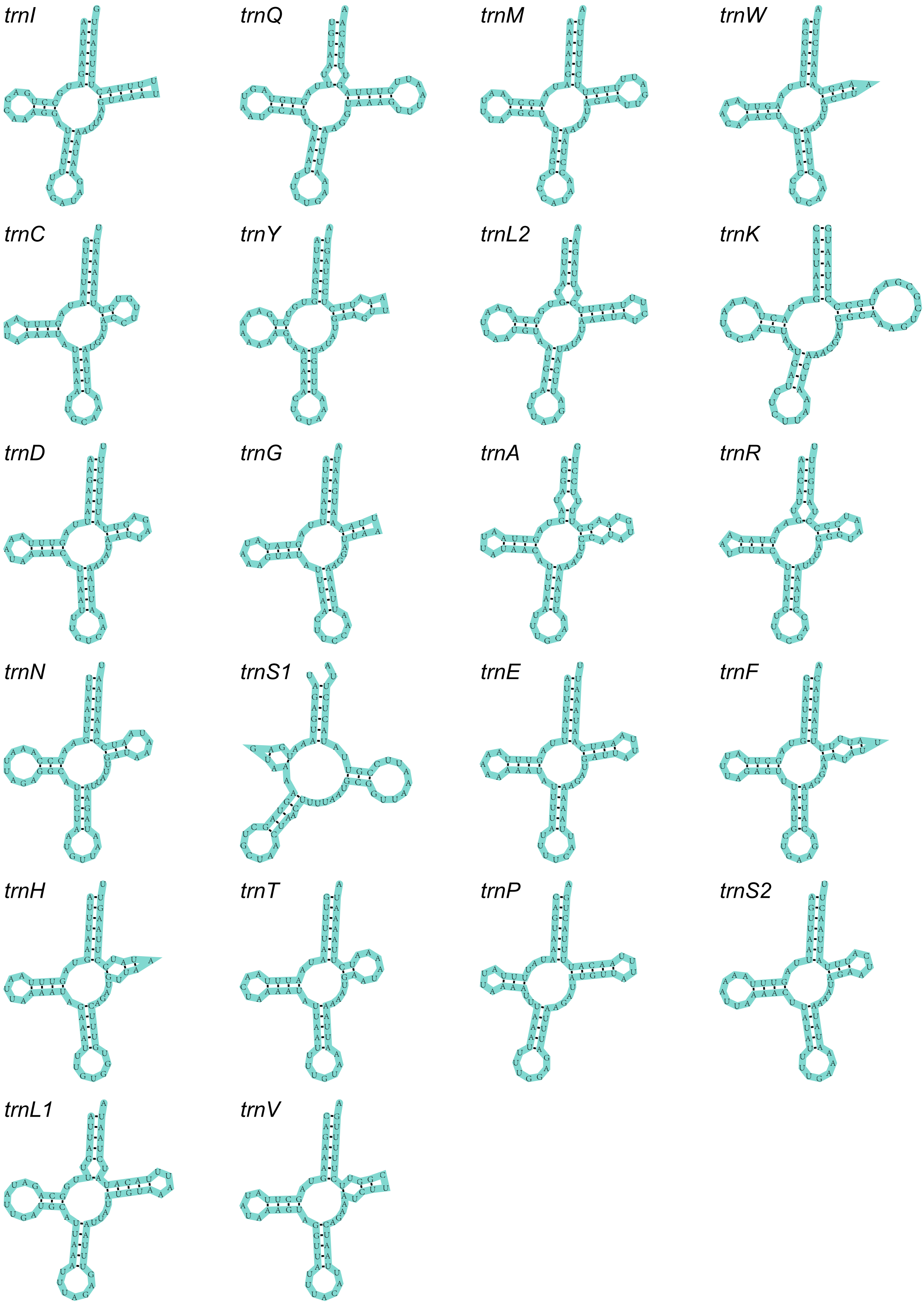

Figure S6. Predicted secondary clover-leaf structure for the tRNA genes of *Nondenticentrus paramelanicus*.

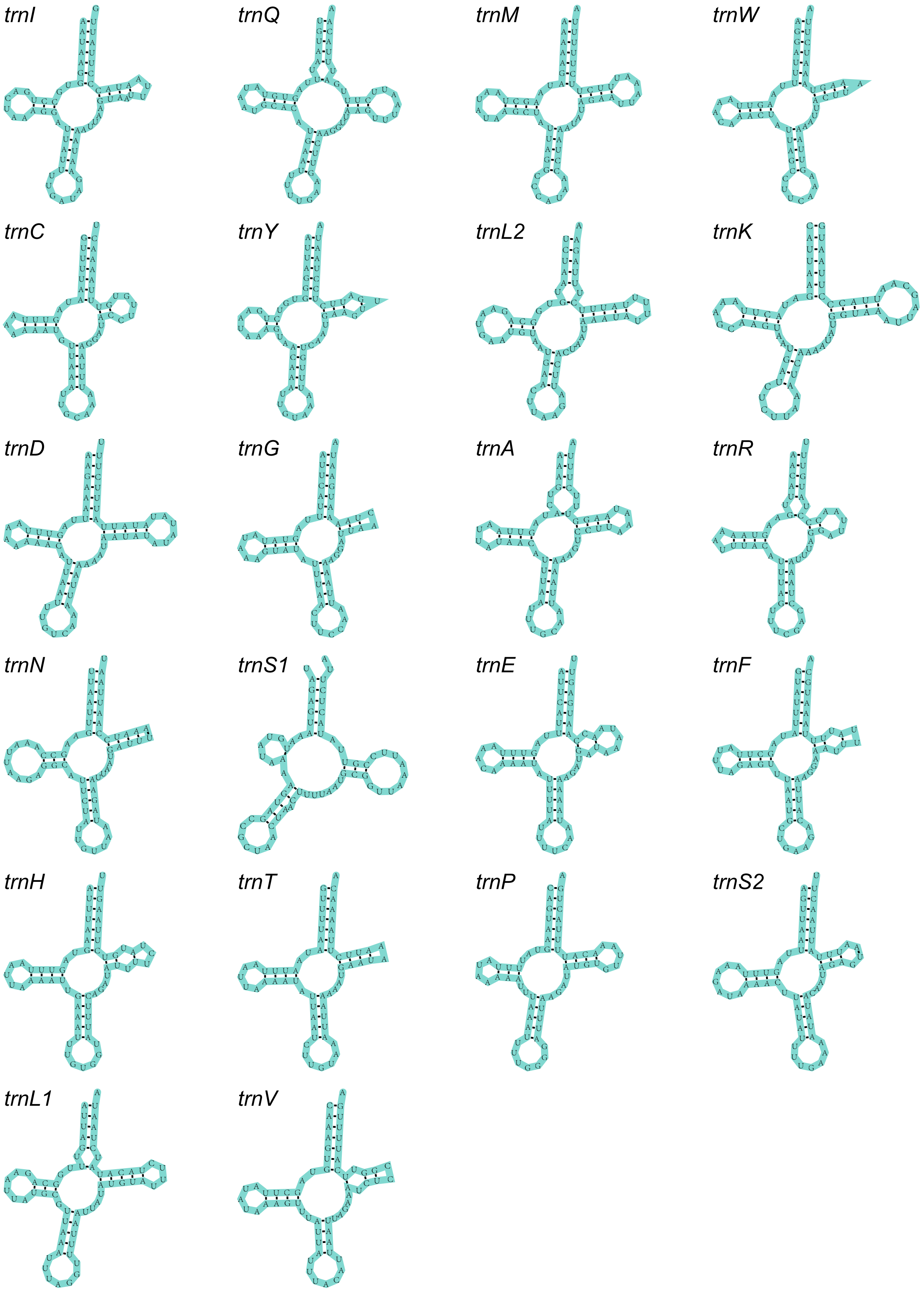

Figure S7. Predicted secondary clover-leaf structure for the tRNA genes of *Pantaleon erectonodatus*.

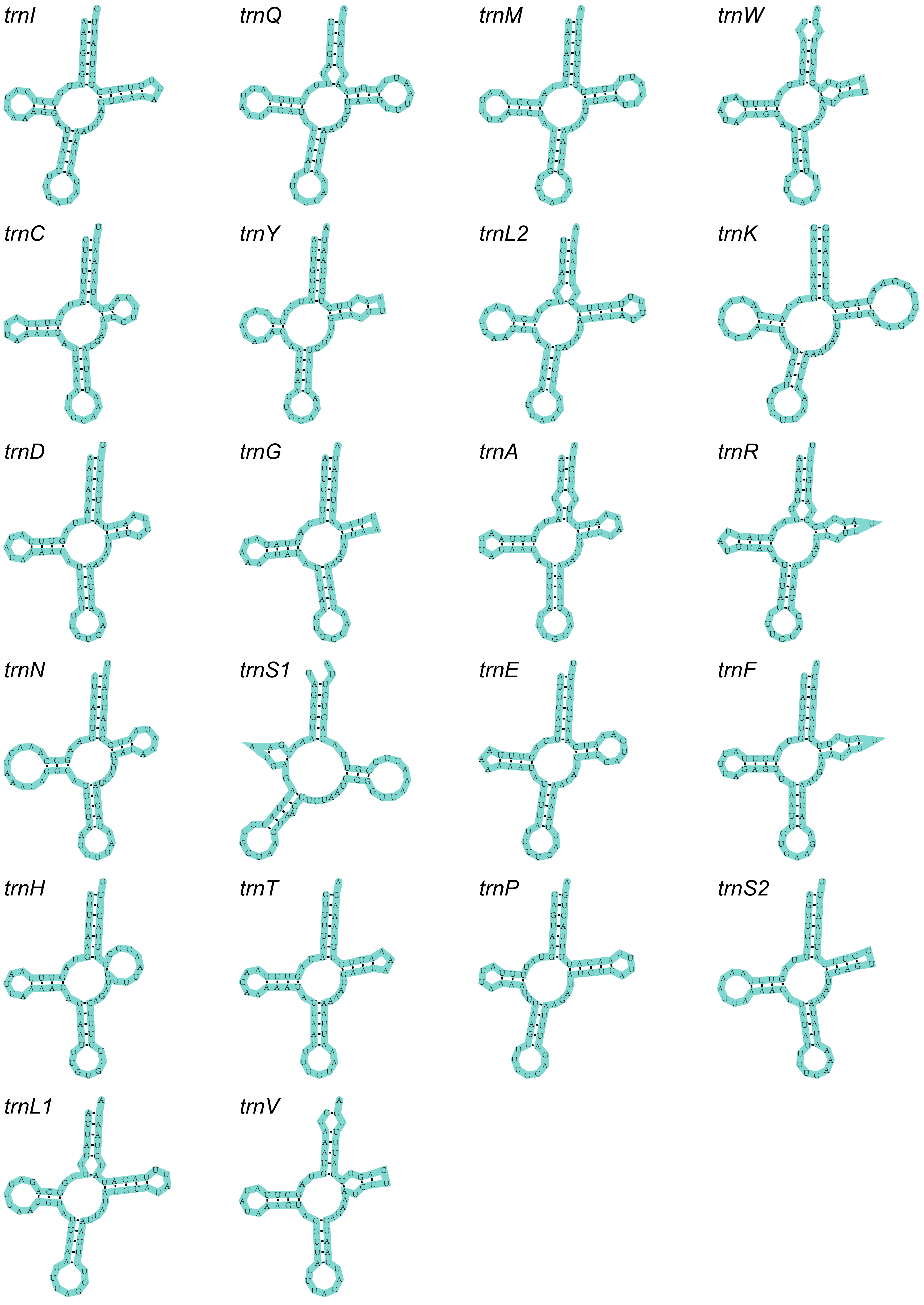

Figure S8. Predicted secondary clover-leaf structure for the tRNA genes of *Tribulocentrus zhenbaensis*.

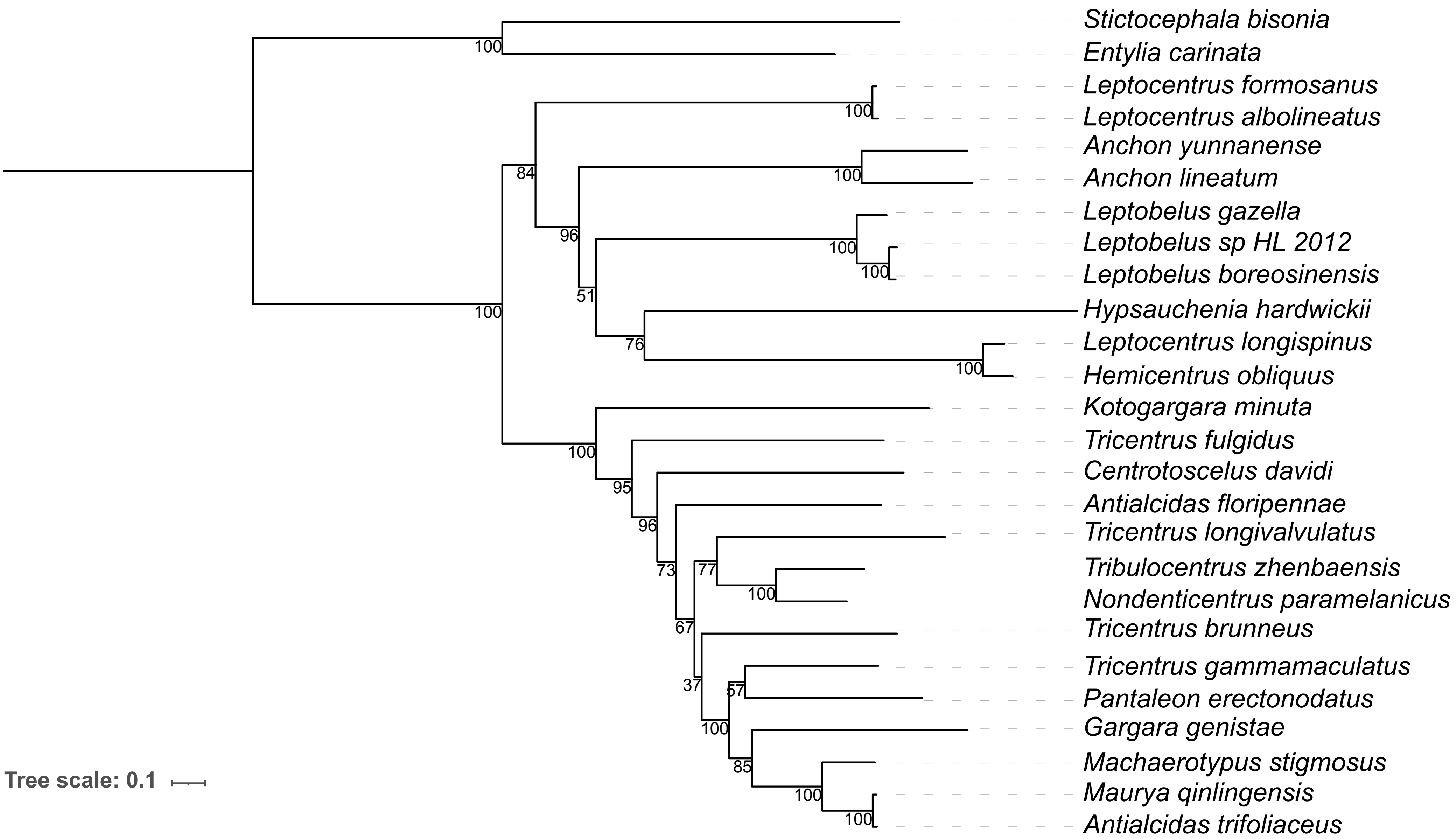

Figure S9. Phylogenetic tree inferred by ML method based on PCG dataset. Numbers on nodes are the bootstrap support values (BS).

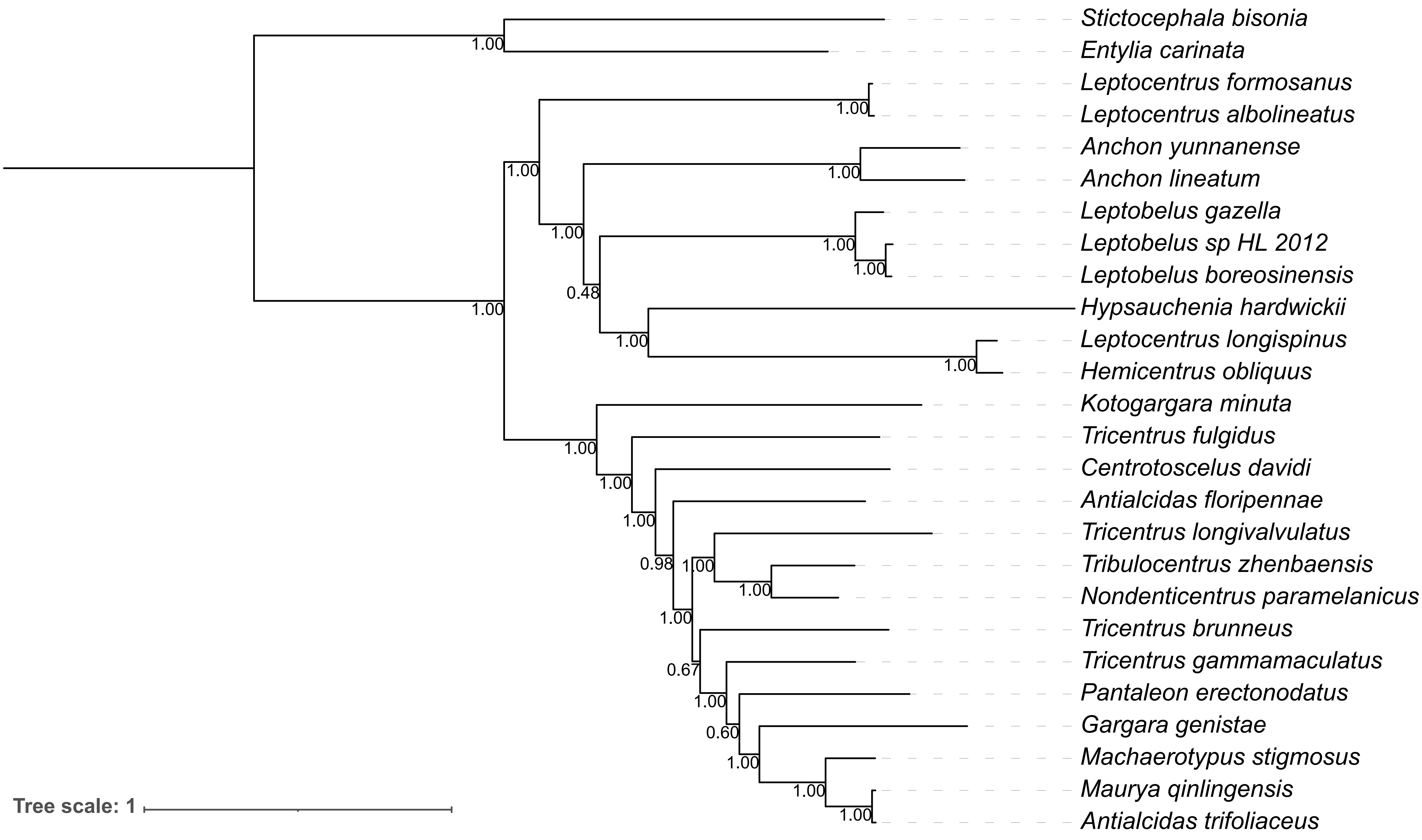

Figure S10. Phylogenetic tree inferred by BI method based on PCG dataset. Numbers on nodes are the posterior probabilities (BPP).

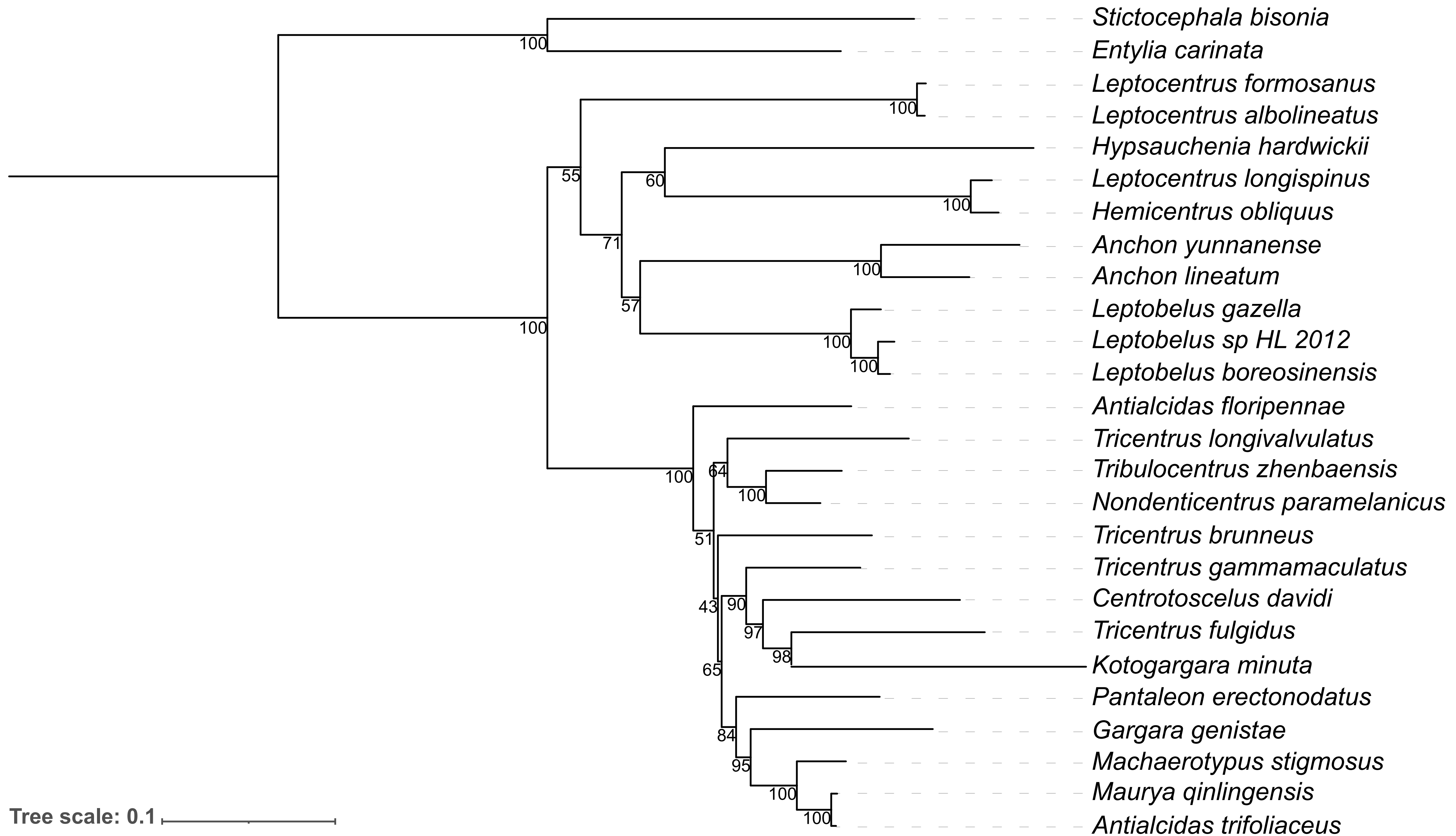

Figure S11. Phylogenetic tree inferred by ML method based on P12RT dataset. Numbers on nodes are the bootstrap support values (BS).

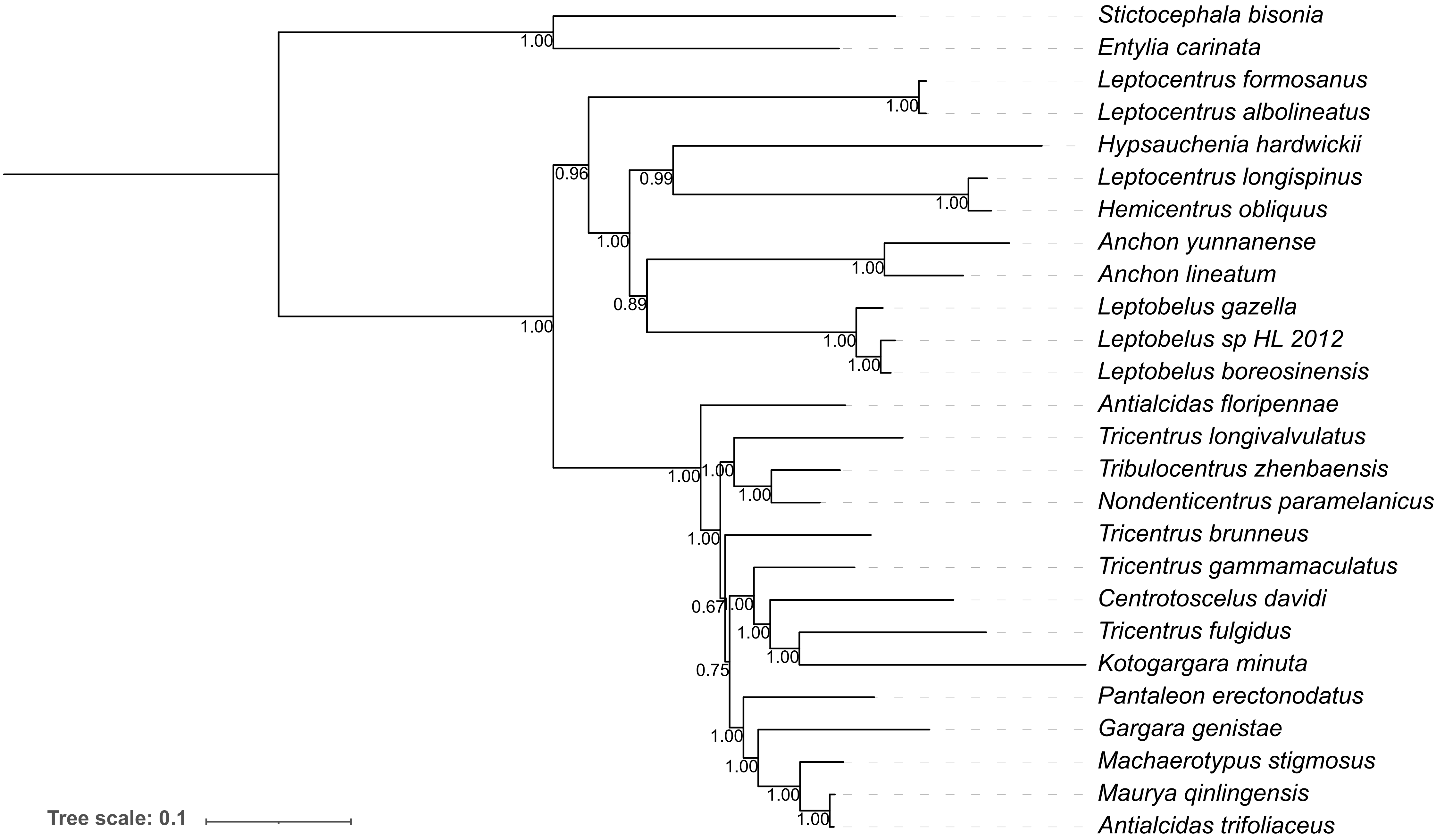

Figure S12. Phylogenetic tree inferred by BI method based on P12RT dataset. Numbers on nodes are the posterior probabilities (BPP).

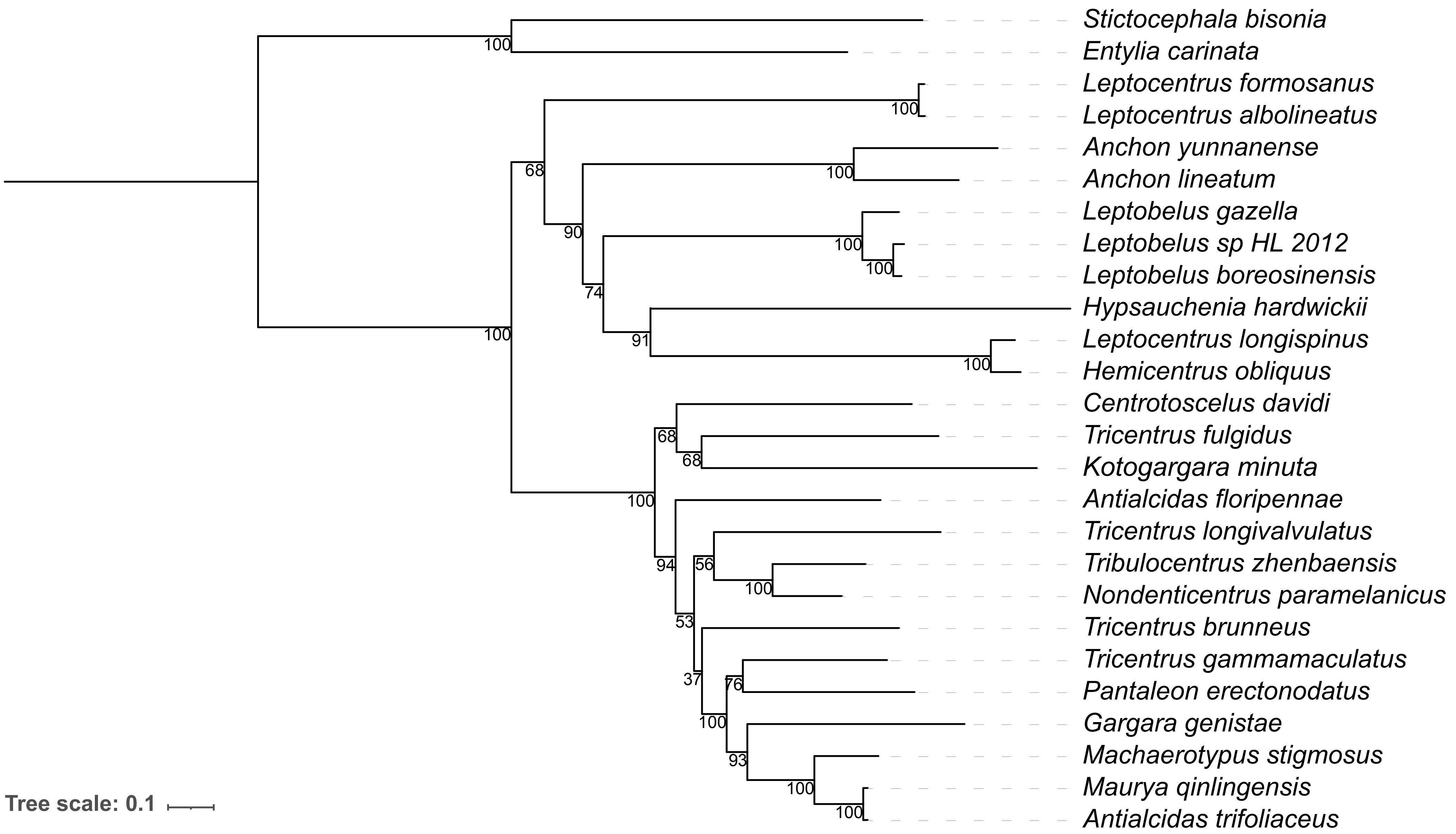

Figure S13. Phylogenetic tree inferred by ML method based on P123RT dataset. Numbers on nodes are the bootstrap support values (BS).
